# Herpes Simplex Virus-Induced Epithelial Damage and Susceptibility to Human Immunodeficiency Virus Type 1 Infection in Human Cervical Organ Culture

**DOI:** 10.1371/journal.pone.0022638

**Published:** 2011-07-27

**Authors:** Julie E. Horbul, Stephen C. Schmechel, Barrie R. L. Miller, Stephen A. Rice, Peter J. Southern

**Affiliations:** 1 Department of Microbiology, University of Minnesota, Minneapolis, Minnesota, United States of America; 2 Department of Laboratory Medicine and Pathology, University of Minnesota, Minneapolis, Minnesota, United States of America; The University of Hong Kong, Hong Kong

## Abstract

Normal human premenopausal cervical tissue has been used to derive primary cell populations and to establish *ex vivo* organ culture systems to study infections with herpes simplex virus (HSV-1 or HSV-2) and human immunodeficiency virus type 1 (HIV-1). Infection with either HSV-1 or HSV-2 rapidly induced multinuclear giant cell formation and widespread damage in mucosal epithelial cells. Subsequent exposure of the damaged mucosal surfaces to HIV-1 revealed frequent co-localization of HSV and HIV-1 antigens. The short-term organ culture system provides direct experimental support for the epidemiological findings that pre-existing sexually transmitted infections, including primary and recurrent herpes virus infections at mucosal surfaces, represent major risk factors for acquisition of primary HIV-1 infection. Epithelial damage in combination with pre-existing inflammation, as described here for overtly normal human premenopausal cervix, creates a highly susceptible environment for the initiation and establishment of primary HIV-1 infection in the sub-mucosa of the cervical transformation zone.

## Introduction

The continuing worldwide HIV/AIDS epidemic is primarily sustained by heterosexual transmission of HIV-1 [1], [2], [3]. Infectious HIV-1 virions and/or HIV-1-infected cells are shed in semen, vaginal secretions, or menstrual blood. Transfer of infectious materials onto mucosal surfaces can result in transmission of HIV-1 into uninfected individuals. Currently, more than half of all newly acquired HIV-1 infections occur in young women following heterosexual exposure. Despite intensive education campaigns and ongoing efforts to develop successful prophylactic vaccination strategies, there has only been modest global impact in reducing current rates of HIV-1 transmission [4]. Animal models of heterosexual HIV-1 transmission, based on vaginal infection of female rhesus macaques, have shown that small foci of infected cells are first detected in proximity to mucosal surfaces within 3–7 days of experimental exposure to SIV [5], [6]. After transfer to the draining lymph nodes, SIV is disseminated via the lymphatic system and the circulation and systemic SIV infection is established in the next 7–21 days [7], [8]. These SIV/macaque results support the fundamental conclusions that protective intervention for HIV-1 must be focused at the site of exposure and must be immediately effective, if HIV-1 replication and spread are to be prevented in recently exposed young women [3], [9], [10].

The mucosal surfaces of the lower regions of the female reproductive tract are comprised of a multi-layered stratified squamous epithelium lining the vaginal cavity and the ectocervix, and a single layer of columnar epithelial cells lining the endocervical canal. Multiple transfer mechanisms, involving uptake by, or infection of macrophages [11], [12] and Langerhans cells [13], and transcytosis by epithelial cells [14] have been proposed to explain how cell-free HIV-1 virions or cell-associated infectious HIV-1 [15], [16], [17], [18] traverse an intact mucosal surface and interact with CD4+ T cells, the principal target cell supporting active HIV-1 replication. We have previously demonstrated rapid and extensive binding and penetration of both HIV-1 virions and seminal cells in *ex vivo* human cervical organ cultures [19] and realized that there may be large elements of variability impacting on the initiation of HIV-1 infection in young women exposed to infectious HIV-1 through heterosexual contacts [20]. From epidemiological studies, it has been recognized that approximately 1 in 200 to 1 in 1000 potential male-to-female exposure events actually results in HIV-1 transmission [21], [22], [23]. This relatively low frequency of transmission can be explained by a continuum of variables extending from the properties of the infectious inoculum to the properties of the exposed mucosal surfaces. The presence of pre-existing sexually transmitted infections (STI) is widely believed to increase susceptibility to HIV-1 infection for exposed women, presumably by causing damage to mucosal surfaces and signaling the recruitment of inflammatory cell infiltrates [24], [25], [26], [27]. Several previous studies have described interactions between herpes simplex virus (HSV-1 or HSV-2) and HIV-1 [28], [29], [30], [31], [32], [33], [34], [35], [36], [37], [38]. In the context of HIV-1 transmission, epithelial cell disruption caused by active herpes virus infections or herpes virus reactivation may create mucosal breaks and expose regions of sub-mucosa where primary HIV-1 infection could readily become established in CD4+ CCR5+ T cells. The presence and distribution of CD4+ T cells as a component of inflammatory infiltrates in the sub-mucosa reflects previous and/or ongoing immunological stimulation. Active STIs, including HSV infections, are known to increase the risk that HIV-1 exposure will progress to primary HIV-1 infection. In this *ex vivo* study, we have infected premenopausal cervical tissue by sequential exposure to HSV-1 or HSV-2 followed by HIV-1 virions in seminal plasma, to develop more specific insight into the cellular and molecular interactions whereby STIs may facilitate the initiation of HIV-1 infection at mucosal surfaces in the normal human female reproductive tract.

## Results and Discussion

### HSV Infection in Primary Epithelial Cells and Fibroblasts

**Figure 1:**
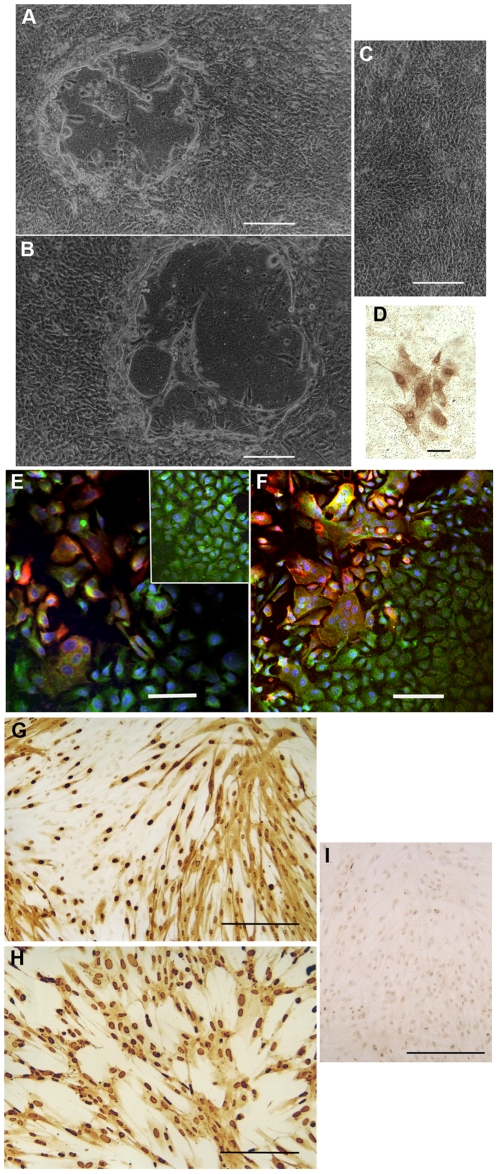
HSV infection of primary human cell populations. Primary human cervical epithelial cells were propagated on transwell membranes and then infected with 1×10^5^ plaque forming units (pfu) of HSV-1 or HSV-2. Localized disruptions in the epithelial cell monolayers were visible after 17 hours for HSV-2 infections and 24–36 hours for HSV-1 infections. A, B: Phase contrast photomicrographs taken at 36 hours post HSV-2 infection. C: Phase contrast photomicrograph of control uninfected primary cervical epithelial cells. D: Detection of HSV-1 antigens by standard colorimetric immunohistochemistry: mouse monoclonal anti-HSV-gB antibody. Brown stain reveals a focus of HSV-1-infected cells at 21 hours post infection. E, F: Detection of HSV-1 antigens at 36 hours post infection by fluorescence microscopy; red: mixture of mouse anti-HSV-ICP4 and mouse anti-HSV-gB monoclonal antibodies, green: rabbit polyclonal anti-cytokeratin antibody, blue: TOTO-3 nuclear stain. Inset in E shows uninfected primary epithelial cells stained with the same antibody mixture. Note also that the lower right corner of F contains a large region of uninfected cells with normal epithelial morphology. Primary human fibroblasts were propagated on glass chamber slides, infected with varying doses of HSV-2 then fixed and processed for standard immunohistochemical detection with a mouse monoclonal antibody directed against HSV-ICP4. G, H: Brown stain reveals extensive HSV-2 infection at 24 hours post infection. Note the presence of some uninfected cells in the upper left area of G and the extensive morphological changes in the infected cell population with 100-fold higher inoculum of HSV-2 in H. Essentially all of the cells were infected with the 1×10^5^ pfu inoculum used in H. I: Control population of uninfected primary cervical fibroblasts that were fixed and processed in parallel with the anti-HSV-ICP4 antibody. Bars A–C = 250 µm; D = 50 µm; E, F = 100 µm; G–I = 250 µm.

In most natural HSV-1 and HSV-2 infections, initial virus replication occurs in epithelial cells at mucosal surfaces [39] and then virus rapidly spreads to sensory ganglia [40], [41], where latent infections become established in the trigeminal ganglia (oropharyngeal HSV-1) or in the sacral ganglia (genital HSV-2). Changes in sexual practices have led to increasing numbers of cases of genital infections now being reported with HSV-1 [31], [42]. Reactivation of latent HSV infection involves virus transport within the sensory ganglia and can result in the (re)-appearance of overt lesions at the original site(s) of infection, or at new sites innervated by the same infected ganglia [40], [41]. Although many experimental HSV infections have used non-human target cells such as immortalized African green monkey kidney cells (Vero cells), some studies have been conducted in human cells, including primary human keratinocytes [43]. HSV infections in epithelial cells are cytolytic [44], [45], as can readily be demonstrated by experimental infection of primary cervical epithelial cells (Fig. 1 A–F). We have also noted cytolytic HSV infections in primary cervical fibroblasts [46], [47] (Fig. 1 G–I). Because of widespread distribution of fibroblasts beneath the cervical epithelium, HSV infection of tissue fibroblasts could have the effect of enlarging foci of damage by providing a secondary target cell population for HSV replication and facilitating HSV entry into peripheral nerve endings [48].

### HSV Infection in *ex vivo* Organ Culture

**Figure 2:**
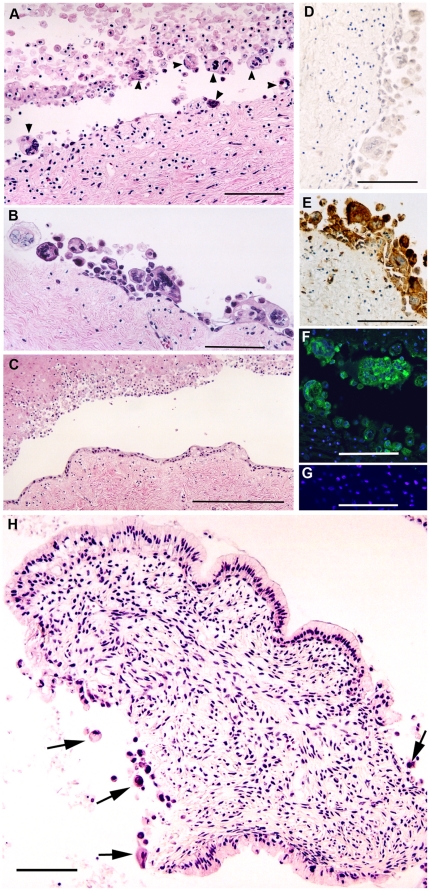
Epithelial damage induced in HSV-infected cervical tissue. Histopathological changes induced in HSV-2-infected ectocervix include areas of extensive epithelial damage with multinucleated giant cells. A, B: Hematoxylin and eosin (H&E) staining of 5 µm tissue sections; pieces of ectocervix were infected with 1×10^5^ pfu of HSV-2 and fixed after 6 days in organ culture. Arrowheads in A indicate multinucleated giant cells – 7 giant cells are visible in ∼500 µm of epithelial surface shown in the figure. C: Control section from an adjacent piece of ectocervix that was incubated *ex vivo* for 6 days without exposure to HSV – some degeneration of the stratified squamous epithelium has occurred but no multinucleated giant cells are present. D, E: Independent sections of ectocervix from the HSV-2-infected tissue shown in panels A and B, processed for standard colorimetric immunohistochemical detection of viral antigens. D: Control antibody directed against HIV-1 p24 gag showing no positive signal (brown stain) despite the presence of multinucleated giant cells. E: Rabbit polyclonal anti-HSV antibody showing strong positive signal (brown stain) in multinucleated giant cells at the disrupted epithelial surface. F: Adjacent section from the HSV-2-infected ectocervix shown in A, B and D, E that was analyzed by confocal fluorescence microscopy; green: rabbit polyclonal anti-HSV antibody, blue: TOTO-3 nuclear stain. G: Control, uninfected tissue processed in parallel with F, using the same antibody mix and the same parameters for fluorescent image capture. H: H&E staining of a 5 µm tissue section of HSV-2-infected endocervix, fixed after 7 days in organ culture. Note the extensive areas of epithelial damage and multinucleated giant cells (arrows). Normal columnar endocervical epithelial cells are visible along the upper and lower surfaces of this tissue section. Bars A, B = 100 µm; C = 250 µm; D–H = 100 µm.

Epithelial cells in both the ectocervical epithelium and the endocervical epithelium were found to be susceptible to infection by herpes simplex viruses. Foci of infected cells, including aberrant giant cells and multinucleated giant cells, were readily observed in tissue pieces exposed to HSV-1 or HSV-2 and frequently coincided with areas of obvious epithelial disorganization (Fig. 2). The pathological changes in *ex vivo* HSV infections very closely resembled changes observed in natural HSV lesions with the characteristic appearance of multinucleated cells with nuclear molding and chromatin margination. When HSV-infected cervical tissue pieces were exposed to fluorescently labeled HIV-1 virions (non-infectious HIV-GFP, [19]), HIV-1 virion binding was readily visible in proximity to HSV-infected epithelial cells (data not shown). These findings were fully consistent with our previous results showing HIV-1 virion binding at sites of epithelial damage when tissue surfaces were deliberately damaged prior to HIV-1 exposure [49].

### HSV and HIV-1 Sequential Infections in Human Mucosal Organ Cultures

**Figure 3:**
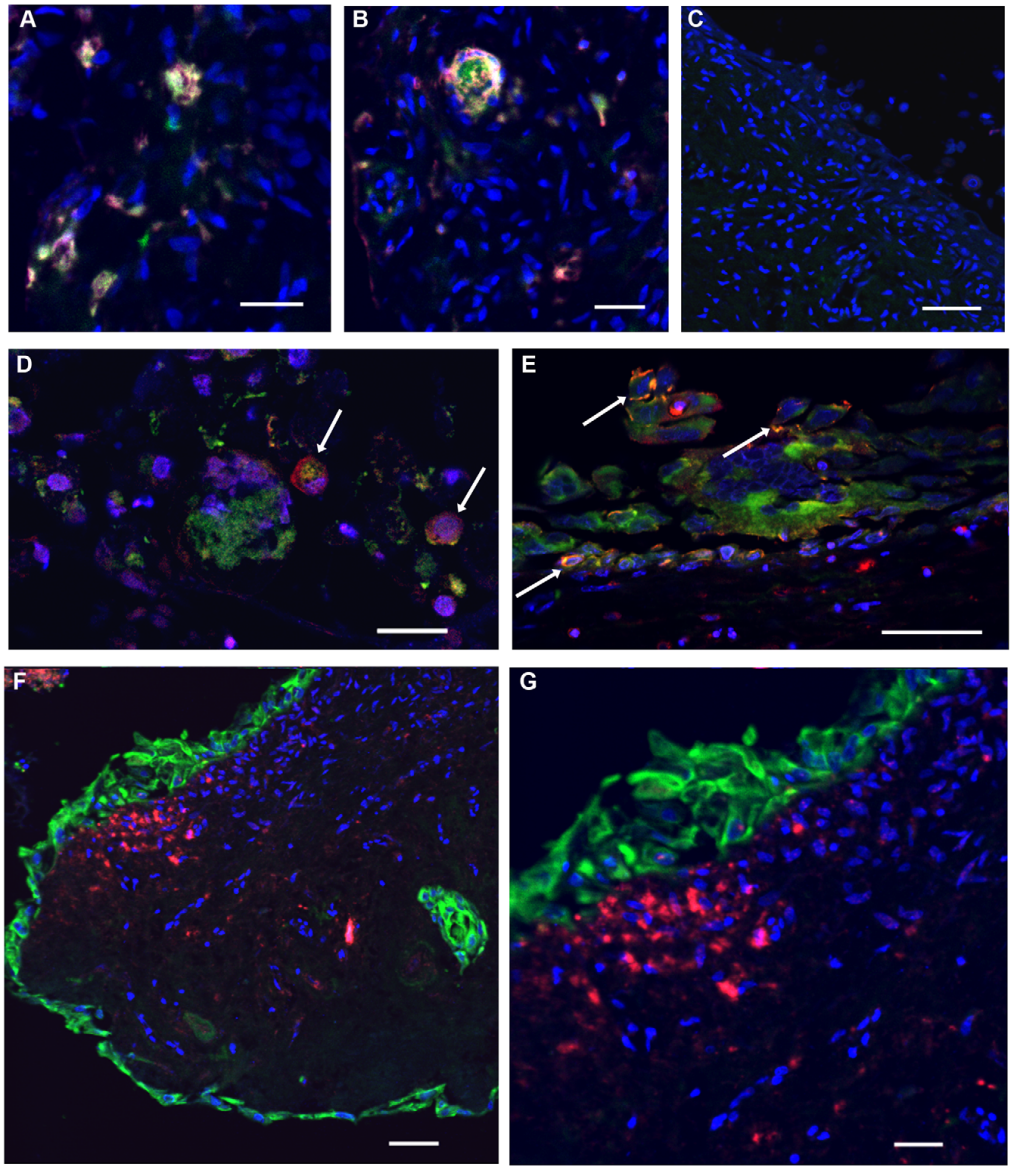
Co-localization of viral antigens in multinucleated giant cells generated from cervical tissue dually-infected with HSV-1+HIV-1. Tissue pieces were infected with 1×10^5^ pfu of HSV-1, incubated for 24 hours then infected with HIV-1 (virus equivalent to 20 pg of p24gag) in normal seminal plasma and then incubated for an additional 120 hours. A, B: Giant cell aggregates visualized by confocal fluorescence microscopy; green: rabbit polyclonal anti-HSV antibody, red: mouse monoclonal anti-HIV-1 p24 gag antibody with TSA enhancement, blue: TOTO-3 nuclear stain. Coincidence of green and red signals produces a yellowish-white color. C: Control uninfected tissue section processed in parallel with the antibody combination used in A, B. Cell nuclei visualized with TOTO-3 nuclear stain. D, E: Detection of HSV-1 and HIV-1 antigens in close proximity at the ectocervical surface; green: rabbit polyclonal anti-HSV antibody, red: mouse monoclonal anti-HIV-1 p24 gag antibody with TSA enhancement, blue: TOTO-3 nuclear stain. Arrows indicate co-localization of HSV-1 and HIV-1 antigens in giant cell forms. F-G: Confocal fluorescence microscopy with normal premenopausal endocervical tissue that was cultured for 6 days prior to fixation and processing; green: rabbit polyclonal anti-cytokeratin antibody detecting epithelial cells, red: mouse monoclonal anti-CD4 antibody, with TSA enhancement, detecting a focus of CD4+ T cells located just below the epithelial surface, blue: TOTO-3 nuclear stain. Bars A = 25 µm; B = 20 µm; C = 50 µm; D = 50 µm; E = 20 µm; F = 50 µm; G = 25 µm.

The basic question of potential interaction between HSV and HIV-1 at mucosal surfaces was examined by sequential *ex vivo* infection of cervical tissue pieces with HSV-1 or HSV-2 followed 24–48 hours later by the addition of HIV-1 virions (dual tropic, low passage patient isolate stock, HIV 96–480 [49]) in seminal plasma. The inclusion of seminal plasma (the cell-free component of normal human semen) in the HIV-1 infection protocol creates a consistent surrogate inoculum that resembles semen from an HIV-1-infected donor. After incubation for 3–5 days to allow the establishment of primary HIV-1 infection, tissue pieces were fixed, embedded in paraffin blocks and sectioned for analysis. Double label immunofluorescence detection of HSV antigens with a rabbit polyclonal antibody and HIV-1 p24 gag with a mouse monoclonal antibody indicated accumulation of viral antigens within multinucleated giant cell aggregates and/or within very closely adjacent cells (Fig. 3 A–E). Tissue sections were also stained to reveal a small inflammatory focus of CD4+ T cells, situated immediately beneath the epithelial surface (Fig. 3 F, G). In this location, any clustering of inflammatory cells may represent a highly susceptible target site for infection wherein HSV-mediated disruption of the surface epithelial layer allows direct access for infectious HIV-1 to the CD4+ T cells in the sub-mucosa. During the progression of HSV infection, cell fusion involving epithelial cells and CD4+ T cells could then form the types of cell aggregates observed to contain both HSV and HIV-1 antigens (Fig. 3A–E). Previous studies ([19] and PJ Southern, unpublished results) have not identified similar multinucleated giant cell forms in *ex vivo* organ culture in the context of HIV-1 infection alone. Additional results relating to the occurrence of pre-existing inflammation and the conspicuous absence of multinucleated giant cells in un-manipulated normal premenopausal cervical tissue are discussed below.

**Figure 4:**
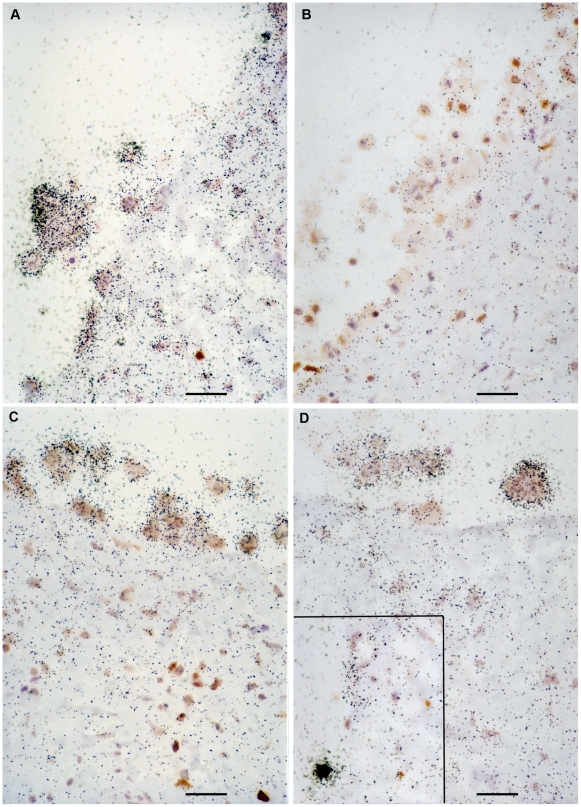
Detection by *in situ* hybridization of HIV-1-infected cell aggregates at mucosal surfaces damaged by prior infection with HSV-2 and immunohistochemical detection of CD3 positive T cells. *In situ* hybridization to detect HIV-1 RNA visualized by the accumulation of black silver grains; CD3 positive T cells visualized with DAB (brown stain); HSV-2 infected cells visualized in the form of multinucleated giant cells. In this experiment, the anti-CD3 antibody detects either CD4+ or CD8+ T cells and both of these T cell populations can be found in proximity to the luminal surfaces in the FRT (see Figure 7 also). A: HSV-2+HIV-1-infected tissue. B: Control tissue infected only with HSV-2, showing minimal accumulation of silver grains. C, D: HSV-2+HIV-1-infected tissue. Inset in D shows a rare cell productively infected with HIV-1. Note that both the density and number of silver grains are higher for the productively infected cell than observed for the multinucleated giant cells. Bar = 25 µm.

As a completely independent approach to examine HIV-1 virion binding and infection at epithelial surfaces damaged by prior HSV infection, we used *in situ* hybridization to detect cytoplasmic HIV-1 RNA as an indicator of productive HIV-1 infection. Clusters of silver grains indicating positive hybridization signal were consistently observed in multinucleated giant cells situated at disrupted epithelial surfaces (Fig. 4). Giant cells and extensive disruption of the endocervical epithelium were not observed in *ex vivo* organ cultures when the initial step of HSV infection was omitted. This cumulative analysis of viral and cellular antigens extends previous work [19] on the cellularity and microarchitecture of cervical mucosal surfaces to highlight the complexity of human mucosal surfaces and the interplay between key parameters affecting susceptibility to microbial infections [20].

There is only limited published information dealing with the susceptibility of human leukocytes to HSV infection but there is evidence for direct HSV infection of CD4+ T cells [50], [51], [52], [53], [54], [55], [56]. In natural infections and, recapitulated here in organ culture experiments, there is the formal possibility that CD4+ cells in the sub-mucosa (T cells, macrophages or dendritic cells) may support dual infections with HSV and HIV-1. However, given the finding of giant cell aggregates expressing both HSV and HIV-1 antigens, the specific issue of dual HSV-HIV-1 infection, within the same cell, is not readily addressed in the experiments reported here. Individual HSV and HIV-1 infections can clearly occur in the absence of the other virus but the independent findings of infected cell aggregates expressing antigens from both viruses and positive HIV-1 *in situ* hybridization signals from multinucleated giant cells provide direct experimental support for a sequential process of HSV-induced damage leading to increased susceptibility to primary HIV-1 infection. Although we have not strictly demonstrated that HSV infections in *ex vivo* organ cultures result in the release of infectious progeny virions, detection of the late HSV protein, glycoprotein B (gB), strongly suggests that the full temporal cascade of herpes virus gene expression is occurring. Furthermore, the morphological appearance of multinucleated giant cells is fully consistent with productive and cytolytic HSV infection.

### Pre-existing Inflammation in Cervical Tissue Samples

**Figure 5:**
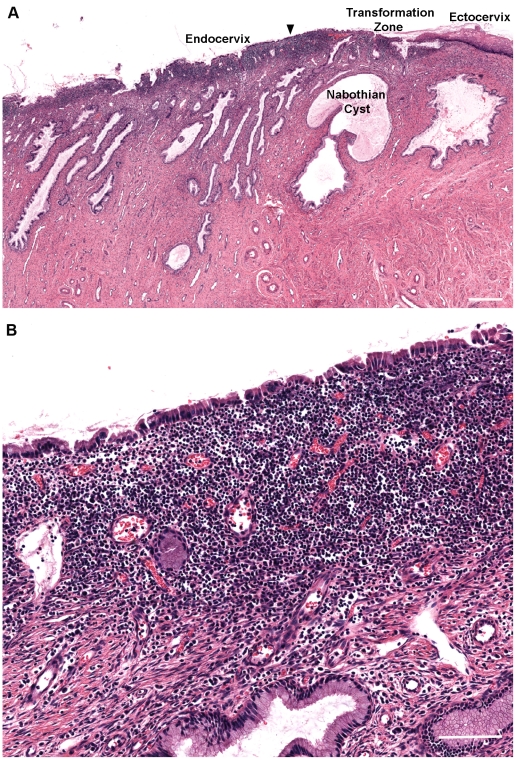
Inflammation at the mucosal surface in human premenopausal cervix. A: H&E staining of the squamocolumnar cervical transformation zone showing extensive inflammation and Nabothian cysts. Bar = 500 µm. Based on the evaluation scheme used for inflammation, this particular tissue was rated: Endocervix: Severe, multifocal, peri-epithelial inflammation. Ectocervix: Moderate, focal, stromal inflammation. In this section, neutrophils, lymphocytes and plasma cells are the primary constituents of the cellular infiltrate. B: Higher magnification image of the endocervical surface (area immediately below the arrowhead in panel A) showing the peri-epithelial location of the inflammatory cells. Bar = 100 µm.

**Figure 6:**
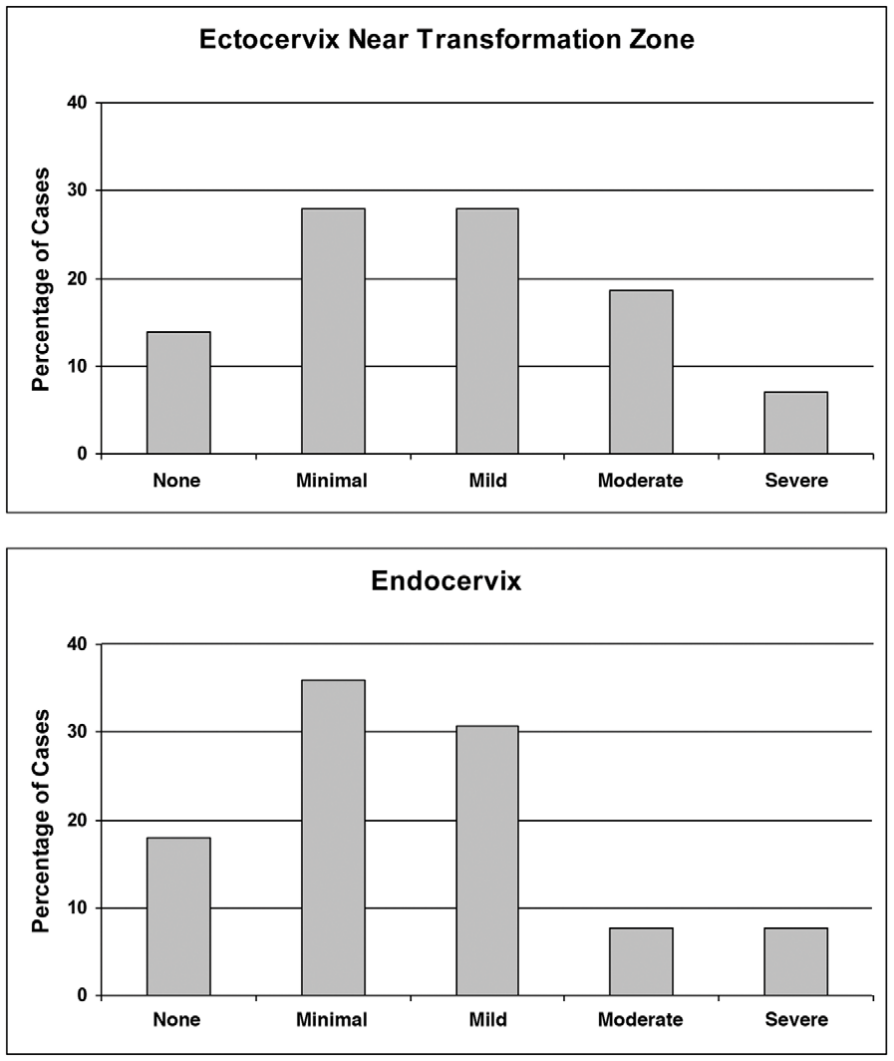
Summary of inflammation observed in the ectocervix in proximity to the squamocolumnar cervical transformation zone and in the endocervix of normal premenopausal women.

In order to develop some awareness of the extent of histological variability that might typically be found in human premenopausal cervix and as a validation of the causal association between acute exogenous HSV infection and the abnormal morphological forms documented in Figures 2, 3, 4, we conducted a retrospective examination of overtly normal cervical samples that were fixed immediately in the Southern laboratory (N = 46; samples collected over a four year period). Foci of inflammatory cells were commonly observed beneath the stratified squamous epithelium of the ectocervix, in proximity to the squamocolumnar transformation zone (23/41 samples with mild-severe inflammation; Figs. 5 and 6) and beneath the simple columnar epithelium of the endocervix (18/39 samples with mild-severe inflammation Figs. 5 and 6). In addition, benign Nabothian cysts were commonly observed in the squamocolumnar transformation zone (22/46 samples; Fig. 5). No multinucleated giant cell complexes were observed in this survey of un-manipulated tissue samples that resembled complexes consistently observed following experimental infections with HSV-1 or HSV-2. However, we did not have access to results for herpes serology evaluations or genital infection diagnostics that might have been performed prior to surgery and so the precise cause(s) of the observed inflammatory foci cannot readily be determined.

The cervical transformation zone is defined by an abrupt transition from the multi-layered ectocervical epithelium to the single cell layer of the endocervical epithelium. In young adult women, the transformation zone is usually located at the lower opening of the endocervical canal (the cervical os). However, around the time of menarche in adolescent girls, the transformation zone usually migrates outwards across the surface of the cervix, well beyond the cervical os, to create a condition known as cervical ectopy [57], [58]. Ectopy is a normal, yet transiently altered condition, with important implications for HIV-1 transmission. Exposed areas of single layer columnar epithelium on the external surface of the cervix may create a highly vulnerable location for HIV-1 infection, especially with coincident inflammation of the sub-mucosa. These changes may account for the particular susceptibility to primary HIV-1 infection that has been observed in adolescent girls [59]. Mild cervicitis is frequently observed in sexually active young women [60], [61], [62], [63] and pre-existing inflammation together with any disruption to the integrity of the epithelial barrier, as may be caused by microbial infections [25], [64], [65] or physical trauma [66], [67], could impact substantially on the initiation of HIV-1 infection [20].

### Immunohistochemical Analysis of Pre-existing Inflammatory Cell Infiltrates

**Figure 7:**
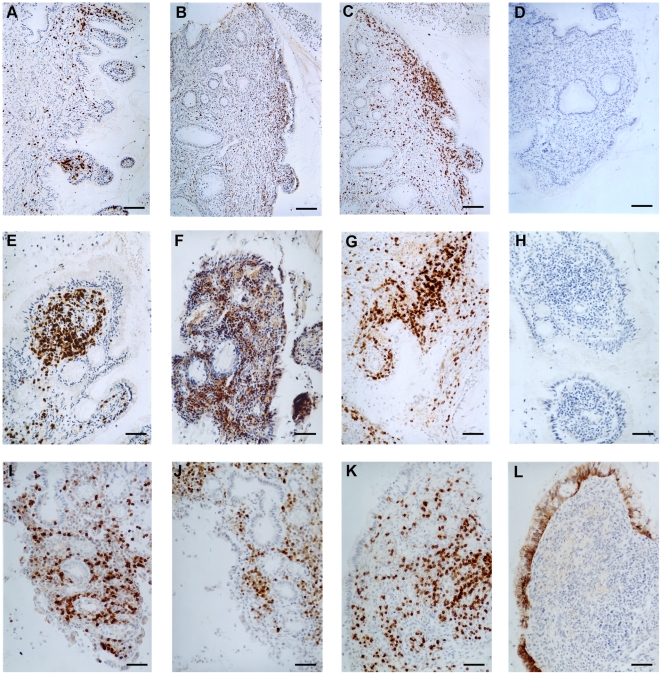
Characterization of inflammatory cell populations in normal human premenopausal endocervix. 5 µm tissue sections were incubated with the primary monoclonal antibodies listed below. Positive signal (brown stain) was revealed with biotinylated secondary antibodies and streptavidin-peroxidase conjugates. Slides were counterstained lightly with hematoxylin. Images are representative of staining patterns observed with 15–20 independent tissue samples. A: CD3. B: CD4. C: CD45RO. D: Control Primary Antibody. A–D from Cervix#1; Bar = 100 µm. E: CD3. F: CD4. G: CD45RO. H: Control Primary Antibody. E–H from Cervix#2; Bar = 50 µm. I: CD3. J: CD4. I–J from Cervix#3; Bar = 50 µm. K: CD3. L: Claudin-4, detecting tight junctions between columnar epithelial cells. K–L from Cervix#4; Bar = 50 µm.

As the first step in understanding the cellular composition and functional capabilities of inflammatory cell infiltrates detected in human cervical tissue, we have used standard immunohistochemical staining to document the presence of CD3, CD4 and CD45RO-positive T cells. Foci of T cells were readily identified in immediate proximity to the endocervical surface (Fig. 7; representative images from the endocervix of 4 different patients) and within and beneath the multilayered stratified squamous epithelium of the ectocervix (not shown). Expression of CD45RO was used as a marker for effector memory T cells located in proximity to a mucosal surface and the presence of this cell population was fully consistent with a recent or ongoing inflammatory event that would have elicited the cellular infiltration. In addition, neutrophils were frequently detected within vessels, distributed throughout the sub-mucosa and trapped in mucus in the endocervical canal (not shown). Because the Institutional Review Board (IRB) approved protocol did not include access to patient medical histories, we were unable to identify specific conditions to account for the inflammatory changes observed (Figs. 5, 6). However, cervicitis is commonly observed in sexually active young women [60], [61], [62], [63] and some component of the inflammation may be triggered by direct contact between seminal plasma and the mucosal surfaces [68].

The presence and distribution of leukocytes in the sub-mucosa reflects extensive vascularization of the endocervix but this same vascularization also provides an immediately accessible conduit to transport HIV-1 infectivity away from sites of initial exposure and primary infection. Based on the proximity of blood vessels and the lymphatic drainage system to the luminal surface of the endocervix and the finding of frequent microlesions in the endocervical epithelium, HIV-1 virions or HIV-1-infected cells in may only have to traverse a distance equivalent to 3–5 cell diameters before encountering a vessel. Dissemination from the portal of entry may therefore be occurring in an overlapping time interval with the formation of sporadic foci of infection in the sub-mucosa. This situation further highlights the protective value of an intact mucosal barrier in impeding HIV-1 transmission and emphasizes the benefits of any available strategies to diminish or eradicate other STIs that are known to disrupt mucosal surfaces.

### Concluding Remarks

The process of HSV-mediated epithelial disruption described here, together with the potential for immediate HIV-1 replication within CD4+ T cells situated in close proximity to the mucosal surface [6], [69] (Figs. 3 and 7), is likely to shift the HIV-1 balance significantly from exposure to establishment of primary HIV-1 infection. Physical damage at epithelial surfaces in female reproductive tissue can occur from either primary HSV infection and/or from HSV reactivation, leading to disruption of the mucosal surface and subsequent inflammation. Although recruitment of inflammatory cells may not be fully reproduced in *ex vivo* organ cultures, it is clear that mucosal damage can be induced in model infections with HSV and that HIV-1 infection can be established in pre-existing CD4+ T cell populations, resident in the exposed tissue pieces [70], [71], [72], [73]. Many STIs are known to be associated with mucosal damage involving either epithelial disruption and/or sub-mucosal inflammation, providing support for the proposal that control of STIs, including HSV [74], [75], [76], could have a beneficial impact on curtailing the spread of new HIV-1 infections. Two epidemiological studies designed to explore connections between HSV and HIV-1 infections [77], [78] appeared to reach conflicting conclusions but this could be explained by differences in patient groups [79]. Amongst sexually active young women, a clear connection was established between HSV-2, or other genital infections, and the risk of acquisition of HIV-1 [78]. Several additional studies have attempted to resolve whether acyclovir therapy to prevent HSV infection and suppress HSV reactivation will have a protective benefit against HIV-1 infection but the trials completed to date have not found any protective effect of daily acyclovir therapy [80], [81]. The overall interpretation of these extensive clinical trials is further complicated by the intriguing finding that phosphorylated acyclovir has an inhibitory effect on HIV-1 replication [82], [83]. Additional trials with acyclovir may have to be conducted. However, at this point in the HIV/AIDS epidemic, the combined recognition that minor histological abnormalities are commonplace in overtly normal premenopausal cervical tissue and that mucosal barrier integrity may be readily disrupted, highlight additional complexities that must be addressed in order to achieve comprehensive mucosal protection against HIV-1 transmission.

## Methods

### Cervical Organ Culture

Normal human premenopausal cervical tissue was obtained within 1–2 hours of completion of the surgery from the BioNet Tissue Procurement Facility at the University of Minnesota Medical Center-Fairview. All tissue donors provided informed written consent, prior to the initiation of surgery, to allow clinical materials to be used for research purposes and all studies were reviewed and approved by the IRB (Institutional Review Board: Human Subjects Committee, Research Subjects' Protection Program, University of Minnesota). Hysterectomies were performed as part of the surgical response to address varying conditions including ovarian carcinoma, uterine fibroids and menorrhagia; tissue from known pathological conditions involving the cervix was not used for any of the *ex vivo* experiments presented here. Some fresh tissue samples were immediately fixed in Streck Tissue Fixative (STF; Streck, Omaha, NE), embedded in paraffin and then processed for routine histological analysis. Cervical inflammation was evaluated according to a mild/moderate/severe and focal/multifocal/diffuse system previously described for human prostate [84]. For both the endocervix and the squamocolumnar cervical transformation zone, inflammation was separately scored for stromal, peri-epithelial and intra-epithelial regions and then condensed to achieve an overall assessment of inflammation ranging from none to severe (Fig. 6). The extent of inflammation was defined as follows:

Minimal: 1–5 inflammatory foci involving ∼10–20 cells

Mild: 1–5 inflammatory foci involving ∼20–50 cells

Moderate: 5–10 inflammatory foci involving ∼20–50 cells and/or foci involving ∼100–200 cells

Severe: diffuse inflammatory foci involving >200 cells.

Tissue samples for experimental infections were either surrounded in agarose/medium wells to leave the mucosal surface exposed or tissue pieces were placed on collagen sponges (Gelfoam, Upjohn/Pharmacia, Kalamazoo, MI), as previously described [19]. Tissue pieces were infected by submersion under a thin film of medium containing the virus inoculum in agarose/medium wells or the virus inoculum was slowly dripped onto the tissue surface from a micropipet tip. In either culture system, tissue surfaces were kept moist by periodic addition of fresh medium, with the effect of washing away any residual unbound virus.

### Primary Cell Culture

Primary populations of cervical epithelial cells and fibroblasts were derived by selective removal of the epithelial surfaces followed by random disruption of the surface cell layer with scalpel blades. In some experiments, epithelial cells were preferentially expanded on 0.4 µm cell culture inserts (transwells, 0.4 µm pore size; Becton Dickinson Labware, Franklin Lakes, NJ) so that the membrane surfaces could be wet on both sides with complete culture medium (RPMI 1640 basal medium supplemented with 10% heat-inactivated fetal calf serum and a standard antibiotic and antifungal mixture - 200units/ml penicillin, 200units/ml streptomycin, 500 ng/ml amphotericin B [Antibiotic-Antimycotic #15240-062; Invitrogen, Carlsbad CA] [19]). Primary cervical fibroblast populations were readily derived from disrupted tissues pieces and these populations could be maintained in continuous culture for 6–10 weeks, using conventional protocols to manipulate adherent cell populations. Epithelial cells and fibroblasts were distinguished based on cell morphology (cuboidal epithelial cells; elongated spindle fibroblasts) and selective high-level expression of cytokeratins by epithelial cells and vimentin by fibroblasts. Primary cell populations were infected either on transwell membranes or after seeding cells onto glass chamber slides. After incubation, washing and fixation, viral and cellular antigens were detected by standard procedures for colorimetric immunohistochemistry or immunofluorescence.

### Viruses

A dual-tropic primary patient isolate of HIV-1 (HIV 96–480; virus stock 200 pg/ml based on p24gag content) was used for all experiments involving infectious HIV-1 [49]. Virion binding studies were performed with non-infectious fluorescently labeled virus particles (HIV-GFP) [19], [49]. Cell-free tissue culture supernatant stocks of laboratory strains of HSV-1 (KOS1.1) and HSV-2 (ATCC Strain G) were prepared by infection of Vero cell monolayers and harvested when extensive cytopathic effects (cpe) were visible in the target Vero cell monolayers. HSV-1 and HSV-2 stocks were used interchangeably for all infections with essentially equivalent results for each virus although HSV-2 infections progressed more rapidly and resulted in more extensive cytopathology. Details in the figure legends identify the particular virus used and the duration of infection. The results shown for HSV infections of primary cell populations, HSV infections of tissue pieces and HSV+HIV-1 dual infections are all representative of independent infections with tissue from 3–5 different donors.

### Antibodies

Primary antibodies to detect cellular or viral antigens were used as described previously [49], [72]. Antibodies were obtained as follows:

HSV glycoprotein B (gB; MAb Catalog #13-120-100; Advanced Biotechnologies Incorporated, Columbia, MD); HSV infected cell protein 4 (ICP4; MAb Clone H1114; Rumbaugh-Goodwin Institute for Cancer Research, Plantation, FL); HSV-1 (Rabbit polyclonal Ab#084P; BioGenex, San Ramon CA); HSV-2 (Rabbit polyclonal Ab#085P; BioGenex); HIV-1 p24 (MAb Clone Kal-1; Dako, Carpinteria, CA); cytokeratins: (Rabbit polyclonal Catalog #Z062201; Dako); CD3 (Rabbit polyclonal Catalog #A045229; Dako); CD4 (MAb Clone 1F6; Zymed Laboratories, South San Francisco, CA); CD45RO (MAb Clone UCHL-1; BioGenex); claudin 4 (MAb Clone 3E2C1: Zymed).

### Immunohistochemistry and Immunofluorescence

Secondary antibodies were either conjugated to biotin for colorimetric detection with a streptavidin-biotin signal amplification system (ABC System, Vector Laboratories, Burlingame, CA) based on horse radish peroxidase and DAB or fluorescent signals were visualized using cy3- and cy5-secondary antibody conjugates (Jackson ImmunoResearch Laboratories, West Grove, PA). Tyramide signal amplification (TSA) was performed exactly as recommended by the manufacturer of the reagents (Renaissance TSA Fluorescence Systems; Perkin Elmer, Boston, MA).

### *In situ* Hybridization and Immunohistochemical Double Labeling

^35^S-labeled antisense riboprobe was prepared by *in vitro* transcription of the *Sac*I linearized plasmid construct containing a 5256 bp fragment of the HIV-1 NL4-3 clone (pNL4-3, generously provided by Dr. Ashley Haase) [85] using T7 RNA polymerase in the presence of ^35^S-rUTP (MP Biomedicals, Solon, OH). After transcription, template plasmid DNA was digested by the addition of RQ1 DNAse (Promega). Labeled RNA probes (∼700 bases) were recovered after DNAse treatment, partial alkaline hydolysis and removal of unincorporated nucleotides using silica affinity membranes (QIAquick Nucleotide Removal Kit, Qiagen, Valencia, CA). Combined *in situ* hybridization and imunohistochemistry was used to detect HIV-1 cytoplasmic RNA and cell antigens on 5 µm deparaffinized tissue sections using previously published methods [5], [72]. Slides were briefly counterstained by dipping in Harris hematoxylin (Fisher), dehydrated and mounted with Permount mounting medium (Fisher).

### Confocal Microscopy

Primary data sets were collected using a BioRad 1024 laser scanning confocal microscope and then processed using Image J, AMIRA and Photoshop software programs.
